# Development, characterization and *In-vitro* evaluation of guar gum based new polymeric matrices for controlled delivery using metformin HCl as model drug

**DOI:** 10.1371/journal.pone.0271623

**Published:** 2022-07-28

**Authors:** Akram Ashames, Kaleem Ullah, Moawia Al-Tabakha, Shujaat Ali Khan, Nageeb Hassan, Abdul Mannan, Muhammad Ikram, Manal Buabeid, Ghulam Murtaza

**Affiliations:** 1 College of Pharmacy and Health Sciences, Ajman University, Ajman, United Arab Emirates; 2 Medical and Bio-allied Health Sciences Research Centre, Ajman University, Ajman, United Arab Emirates; 3 Department of Pharmacy, COMSATS University Islamabad, Abbottabad, Pakistan; 4 Hamdard Institute of Pharmaceutical Sciences, Hamdard University, Islamabad, Pakistan; 5 Department of Pharmacy, COMSATS University Islamabad, Lahore, Pakistan; Universiti Malaya, MALAYSIA

## Abstract

Currently, hydrogels are considered as ideal biomaterials due to their unique structure and characteristics that facilitates considerable hydrophilicity, swelling, drug loading and release. In this study, we report pH-responsive GG-MAA-AMPS hydrogel delivery system prepared via free radical polymerization technique. Hydrogels were loaded with Metformin HCl as a model drug. Hydrogels were characterized through Fourier transform infrared spectroscopy (FTIR), thermogravimetric analysis (TGA), differential scanning calorimetry (DSC), X-ray diffraction (XRD) and scanning electron microscopy (SEM). FTIR confirmed the successful crosslinking of reactants, hydrogel network formation and drug loading. TGA and DSC proved the higher thermal stability of reactants after crosslinking and drug loading. XRD analysis showed decrease in crystallinity of drug after loading into the hydrogels. SEM revealed smooth and glassy appearance of both loaded and unloaded hydrogels. Gel content was increased with increase in concentration of reactants. Drug entrapment was decreased by increasing concentration of GG and AMPS while MAA acted inversely. Hydrogels displayed pH-dependent swelling and drug release behavior being high at pH 6.8 and 7.4 while low at acidic pH (1.2). Oral tolerability in rabbits showed that hydrogels were safe without causing any hematological or histopathological changes in healthy rabbits. Based on the obtained results, GG-MAA-AMPS can be considered as potential carrier for metformin HCl as well as other hydrophilic drugs.

## 1. Introduction

Polymers based oral-colonic delivery of various drugs have been widely explored in recent past. Stimuli responsive hydrogels termed as “smart hydrogels” are considered as cornerstone of polymer based drug delivery systems. Smart hydrogels are decorated with the potential to deliver and release the drug at desired site and rate. Hydrogels are class of soft materials crosslinked physically or chemically with defined 3-dimensional polymer network structure. They are highly swellable, absorb large quantities of water as well as biological fluids and remain undissolved. They resemble soft tissues in swollen state. Polymers used in smart hydrogels for oral-colonic delivery possess weakly acidic groups so they remain shrinked and unswollen in acidic environment of upper gastrointestinal tract (GIT) and stomach (pH 1.2). However, they exhibit significant swelling in lower part of GIT and more specifically in colon where pH is high (7–7.5) [1,2].

Multi-polymer based smart hydrogels are more in practice because they provide with the advantage of tenability and efficient drug delivery compared with single polymer based hydrogels. The divergence in applications cannot be fulfilled by homo and co-polymeric hydrogel system, therefore, researchers are showing their large interest in interpenetrating network (IPNs) systems [3].

The IPNs are comprised of two or more than two polymeric networks where one polymeric system is fabricated in the presence of the other. Semi-IPNs are systems where one polymer is linear while the other is cross-linked. IPNs are more favored in drug delivery and biomedical applications due to enhanced swelling profile, efficient drug entrapment and comparatively dense, denser and stiffer mechanical characteristics compared with the conventional hydrogel systems [4,5].

Natural polysaccharides based smart hydrogels are more advantageous and interesting due to their renewable character, biodegradability and low cast [6]. Among natural polysaccharides, guar gum is most widely used in smart hydrogels for oral-colonic delivery of various drugs. Guar gum is a naturally occurring polymer primarily obtained from seeds of Cyamopsis tetragonolobus [7]. In recent past, guar gum and its derivatives are used as most promising material in pharmaceutical, biomedical and environmental fields due to interesting characteristics such as biodegradability, bioavailability hydrophilicity and non-toxicity [8]. GG was used in current study due to its functional properties of controlling the drug release in gastro-intestinal tract (GIT) specifically for oral colon targeted drugs [9,10], for efficient delivery of anticancer drugs in colorectal cancer (CRC) [11], for oral rehydration solutions in the treatment of adult’s cholera [12], transdermal drug delivery [13] and wound healing [14].

2-acrylamido-2-methylpropanesulfonic acid (AMPS) is a hydrophilic monomer that contains anionic and also non-ionic groups. It is an ionic co-monomer of acrylamide (AAm), and widely used in drug delivery systems due to the presence of strongly ionizable sulfonate group (SO_3_H). AMPS completely dissociates across the overall pH range and therefore the hydrogels derived from AMPS exhibit pH independent swelling behavior [15].

In current study Metformin HCl was used as model drug. It belongs to Biguanide with anti-hyperglycemic and insulin-sensitizing properties. Metformin-HCl release kinetics have proved its slow and incomplete absorption (50–60% bioavailability). After oral administration 30–50% is excreted unchanged in urine within 24 hrs while approximately 30% is excreted in feces. Most common side effects related with metformin therapy includes diarrhea, dyspepsia, nausea, flatulence and abdominal pain [16,17]. Keeping in view the stated problems, metformin formulations have been developed to increase adherence, reduce side effects and control the drug release.

In this study, we report the development of pH-responsive hydrogels based on crosslinking of GG-AMPS-MAA through free radical polymerization technique. Metformin HCl was selected as model drug because it needs a controlled delivery systems to ensure its target specific release. The prepared hydrogels were characterized through FTIR, XRD, TGA, DSC and SEM. The hydrogels were evaluated for sol-gel and drug entrapment efficiency. Swelling and drug release studies were performed at various pH conditions. In-vitro drug release mechanism was determined by applying various kinetic models on drug release data. *In-vivo* biocompatibility and oral tolerability was tested in rabbits.

## 2. Experimental

### 2.1 Materials

Guar Gum (GG), methylene bisacrylamide (MBA) and ammonium persulfate (APS) were purchased from Sigma Aldrich (Germany). Methacrylic acid (MAA) was purchased from BDH, (UK) while 2-acrylamido-2-methylpropanoesulfonic acid (AMPS) was purchased from Daejung (Korea). Potassium chloride (KCl), sodium hydroxide (NaOH), potassium dihydrogen phosphate (KH_2_PO_4_) and hydrochloric acid were purchased from Merck (Germany). Metformin HCl was kindly gifted by Ferozsons Laboratories Limited, Nowshera, Khyber Pukhtunkhwa (Pakistan).

### 2.2 Synthesis of GG-AMPS-MAA pH-responsive hydrogels

**Fig 1:**
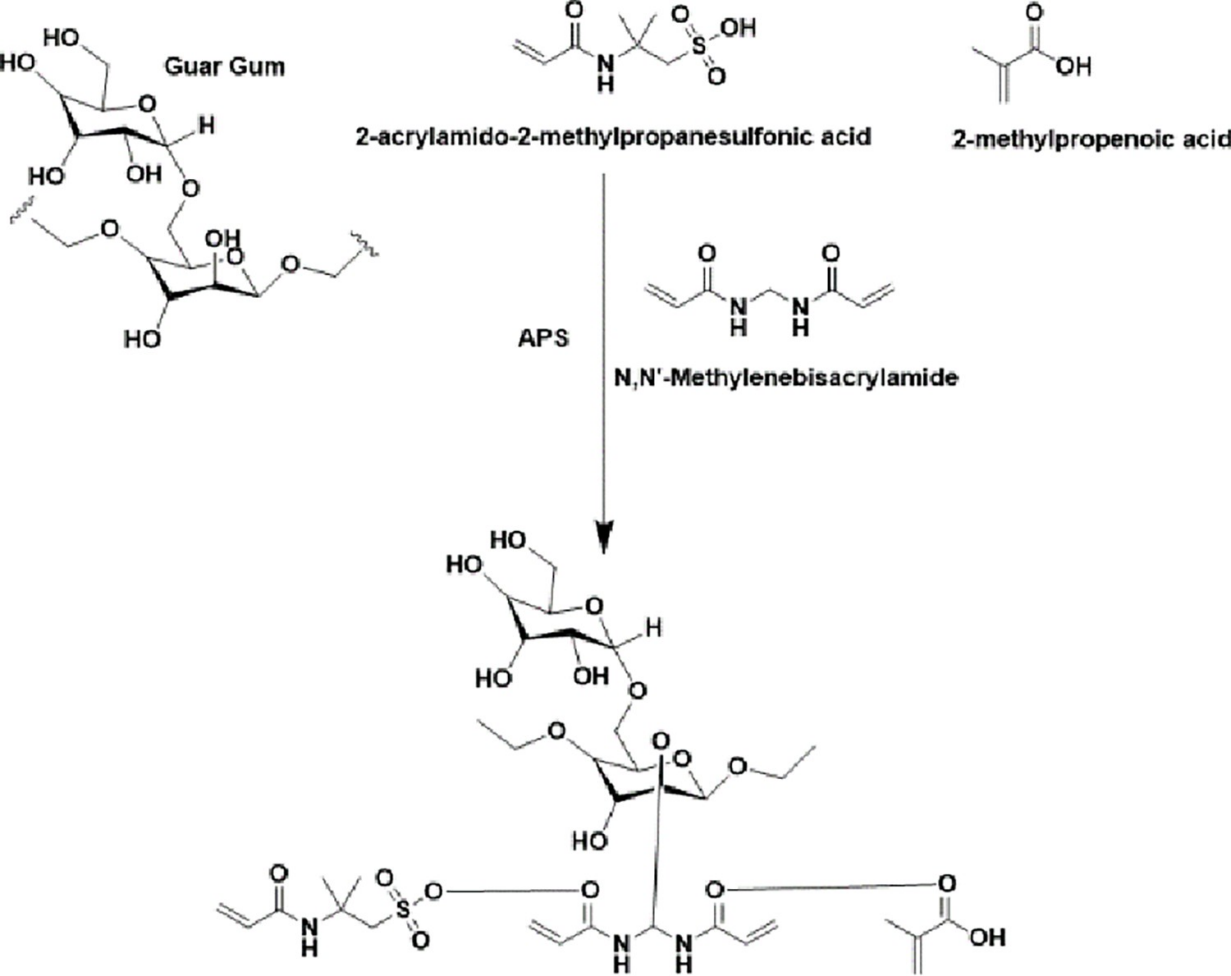
Proposed structure of GG-AMPS-MAA hydrogels.

In our study, previously reported free radical copolymerization technique was used for the synthesis of GG-AMPS-MAA hydrogels (Fig 1) with slight modifications [18]. In brief, specific quantities (Table 1) of GG, AMPS, APS (reaction initiator) and MBA (crosslinking agent) were weighed and dissolved in distilled water with continuous stirring using hot plate magnetic stirrer at 2000–3000 revolution per minute (rpm). Thereafter, solution of APS was slowly added to methacrylic acid and allowed to stir for 30 min. followed by addition of AMPS solution. This mixture was kept on stirring for 45 min to form a clear solution. The resultant clear solution was slowly mixed with the already prepared GG solution and again stirred for 20 min. This solution now contain AMPS, MAA, GG and APS. Lastly, MBA solution was added drop by drop with continuous stirring for 30 min. at 6000–7000 rpm. The final clear solution was carefully filled in pre-labelled glass test tubes having 8 mm diameter and covered with aluminum foil to avoid escape of moisture. The test tubes were kept in water bath at 45°C for 2 hrs. then 50°C for next 4 hrs. and lastly at 55°C for 24 hrs. Gel formation was noticed within initial 6 hrs. of incubation. The newly formed transparent hydrogels were carefully washed with ethanol-water mixture (30:70) separately, blotted with filter paper and cut into 8 mm diameter discs. A portion of each formulation was left unwashed for sol-gel studies. Initially the washed hydrogel discs were dried at room temperature for 24 hrs. followed by placement in oven at 40°C for 72 hrs. to complete drying. The completely dried hydrogel discs were stored in pre-labelled air tight glass containers for various studies.

**Table 1:** Formulation sheet.

S. No	Formulation code	Guar Gum gm/100 gm	MAA gm/100 gm	AMPS gm/100 gm	MBA gm/100 gm	APS
1	GG1	0.1	12	8	0.2	1
2	GG2	0.2	12	8	0.2	1
3	GG3	0.3	12	8	0.2	1
4	MA1	0.1	15	8	0.2	1
5	MA2	0.1	18	8	0.2	1
6	MA3	0.1	21	8	0.2	1
7	AP1	0.1	21	10	0.2	1
8	AP2	0.1	21	12	0.2	1
9	AP3	0.1	21	14	0.2	1

### 2.3 Metformin HCl loading

Metformin HCl was loaded in the newly formed hydrogels following swelling and diffusion method. The drug solution at 1% concentration was prepared using phosphate buffer pH 7.4 maintained at 37°C. Dried hydrogel discs of each formulation were immersed in 100 mL of drug solution. Disc position was changed every 6 hrs. to ensure uniform exposure of hydrogel discs to the drug solution. This process was continued for 96 hrs. followed by careful removal of the swollen hydrogel discs from the jars. Initially, the drug containing swollen hydrogel discs were dried at room temperature followed by oven drying at 40°C until constant weight was attained [19].

### 2.4 In-vitro characterization

#### 2.4.1 Fourier transform infrared spectroscopy (FTIR)

FTIR was performed for polymer, monomers, drug, unloaded and drug loaded hydrogels. The analysis was performed to determine the physical or chemical interaction between polymer and monomers, successful network formation and crosslinking as well as loading of drug in hydrogel network using Tensor 25 series FTIR (Germany). Approximately 15 mg finely crushed sample was scanned in the wavelength range between 4000–500 cm^-1^. Spectral analysis approach was adopted for interpretation of results [18].

#### 2.4.2 X-ray diffraction analysis (XRD)

Chemical crosslinking, physical interaction and drug loading may affect the crystallinity of polymer, monomers or drug. Hence XRD analysis was performed to determine any change in crystallinity profile of reactants and drug after crosslinking and loading of drug in hydrogels. The analysis was performed for drug, unloaded and drug loaded hydrogel. XRD patterns were obtained using PAN analytical diffractometer (X’pert Pro, UK) equipped with copper (Cu) radiation source (ʎ 1.542 Å) and voltage source of 40 KV. The samples were analyzed in the range of 10–100° at 2°/min scan rate [4].

#### 2.4.3 Thermo-gravimetric analysis (TGA)

The analysis was performed in order to investigate the change in thermal profile and weight loss after hydrogel network formation and drug loading. For analysis, 10 mg hydrogel sample was finely grounded and taken in aluminum pan. The instrument (Q-500 series, WestSussex, UK) was operated in standard mode and sample was heated in the range of 20–500°C at 10°C/min temperature increment and 50mL/min nitrogen purging. Universal Analysis 2000 (version 4.5A) software was used to collect and interpret the data [20].

#### 2.4.4 Differential scanning calorimetry (DSC)

DSC was performed to investigate the glass transition temperature (*Tg*) and melting temperature (*Tm*) of the drug, polymer, monomers, unloaded and drug loaded hydrogel. For this purpose, approximately 5 mg finely grounded sample was placed in an aluminum pan and heated in the temperature range between 20–500°C under standard temperature mode. Temperature was accelerated at 10°C/min while nitrogen purging was set at 50 mL/min. For analysis, Q-2000 series, WestSuccex, UK instrument was used while for data collection and interpretation, Universal Analysis 2000 (version 4.5A) software was used [21].

#### 2.4.5 Scanning electron microscopy (SEM)

Scanning electron microscopy was performed for the surface and cross-sectional morphology of unloaded and drug loaded hydrogels. VEGA 3, TE SCAN, Czech Republic SEM apparatus was used. Prior to analysis, each sample was gold plated in order to achieve better conductivity. The sample was mounted on aluminum stub using double adhesive tape. The instrument was operated in open mode and photomicrographs were captured at various magnifications [2].

## 2.5 Sol-gel analysis

Sol-gel analysis was performed to calculate un-reacted contents in newly formed hydrogels. Soxhlet extraction technique was used for this purpose. Oven dried unwashed hydrogel discs were carefully weighed and added to soxhlet apparatus containing de-ionized boiling water. Hydrogel discs were removed after 4 hrs, blotted with filter paper and subjected to drying until constant weight was attained [22]. Following equation was used to calculate the sol fraction and gel fraction separately.

$$Gel\;fraction=Mi-\frac{Me}{Mi}\times 100$$

Where, Mi represents the initial weight of dry gel, Me represents the weight of gel after extraction.


Solfraction=100‐Gelfraction

## 2.6 *In-vitro* swelling studies

The pH responsive behavior of the prepared hydrogels was determined at pH 1.2, 6.8 and 7.4 to simulate stomach, intestinal and colonic conditions respectively. Pre-weighed, oven dried hydrogel discs were immersed in 100 mL of respective pH solutions at 37°C. Hydrogel discs were taken out at regular pre-defined time intervals, blotted with filter paper to remove the excess buffer and then weighed. The process was continued until the equilibrium weight was attained [22]. The study was performed in triplicate while special emphasis was on maintaining the pH of the buffer solution on daily basis.

Following equation was used to determine the swelling index:

$$\%SR=\frac{\left(Ws-Wd\right)}{Wd}\times 100$$

Where,

Ws is weight of swollen hydrogel disc, and Wd is weight of initially dried disc

### 2.7 Estimation of loaded drug

For estimation of the loaded drug, extraction and solvent replacement method was followed as reported earlier. The drug loaded hydrogel disc was immersed in pre-labelled beakers containing 25 mL of PBS pH 7.4 [19]. The buffer was replaced every 24 hrs and analyzed at 235 nm using UV-spectrophotometer (PG instrument, T80+ series, UK). The process was continued until constant absorbance values were obtained.

### 2.8 *In-vitro* drug release studies

The study was performed to investigate pH-responsive behaviour of the hydrogels as well as effect of varying concentrations of polymer and monomers on *in-vitro* drug release. The study was performed at pH 1.2 (0.1N HCl), 6.8 and 7.4 (USP-Phosphate buffer solution) using USP dissolution apparatus-II (Microtek Digital, Pak). The experimental conditions were maintained at 37° ± 0.5°C and 100 ± 5 rpm containing 900 mL dissolution media. Drug loaded, oven dried hydrogel disc was immersed in each basket. Sample (5 mL) was withdrawn at pre-determined time points and replaced with fresh buffer to ensure the sink conditions [18]. The withdrawn sample was analysed at 235 nm using UV-spectrophotometer.

### 2.9 Analysis of drug release data using model dependent approach

For determination of metformin HCl release mechanism, *In-vitro* drug release data was evaluated by using various models like Zero-order, First-order, Highuchi and Krossmyer-Peppas model.

### 2.10 *In-vivo* safety profiling using rabbit model

The study was performed following guidelines suggested by Laboratory Animal’s Science Association [23].

#### 2.10.1 Ethical approval

The study decorum was critically evaluated by Research Ethics Committee, COMSATS University Islamabad, Abbottabad Campus (Pakistan) to ensure all the ethical, safety and hygienic parameters. The suggested decorum was approved and granted ethical approval number Phm.Eth/FA17-CSM10/18-010-74.

#### 2.10.2 Animal Procurement and acclimatization

Initially, 16 rabbits (8 male, 8 female) having average 2–2.2 kg body weight were procured. All the rabbits were placed in wooden boxes with free access to food and water. During this period, the rabbits were closely observed for signs of food and water intake, salivation, urination, response to sound and light, illness and death for a period of 1 week.

#### 2.10.3 Final selection of animals, MTD calculation and administration

Hydrogel suspension was administered following maximal tolerance dose (MTD) method as no lethal dose (LD_50_) dose could be determined. For MTD calculation, 4 rabbits (2 male and 2 female) were selected. Hydrogel suspension was prepared in 15 mL distilled water and administered using 50 cc syringe connected to feeding tube. The suspension was administered in the range of 400–3800 mg/kg of body weight in 2 divided doses. The concentration of hydrogel was increased at a frequency of 100 mg/kg of body weight while volume of water was kept constant. For oral tolerability, 12 rabbits (2–2.2 kg average body weight) were selected and divided into 2 groups each congaing 6 rabbits (3 male and 3 female). The rabbits were properly labeled and housed in separate cages. Group-I was named control while group-II treatment group. Prior to administration of initial dose, all the rabbits were kept fasted for 15 hrs. while free access to water was granted. Group-I was administered normal saline while group-II was administered 3600 mg/kg of body weight hydrogel suspension in 2 divided doses. The study continued for seven days and during this period rabbits were critically observed for food and water intake, urination, salivation, motor activities, illness and mortality. On day 8^th^, blood samples were collected from jugular vein of each rabbit and preserved in EDTA tubes. A portion of each blood sample was left clotted. Later, all the animals were sacrificed under hygienic conditions and organs were carefully separated without causing any damage. The separated organs were preserved in pre-labelled glass jars containing 100 mL 10% formaldehyde (buffered) to ensure complete immersion. Thereafter, the blood samples (clotted and un-clotted) and organs samples were transferred to department of pathology, Ayub medical complex (Abbottabad, Pakistan) where hematology, serum chemistry and pathology studies were conducted under the supervision of consultant pathologist.

### 2.11 Statistical analysis and software used

The data in triplicate is presented as mean ± standard deviation (SD). T-test was applied on sol-gel, swelling, drug loading and release data. ANOVA was applied for comparison of multiple groups while for statistical significance, *P-*value ≤ 0.05 was assumed as significant. All the images and graphs were drawn using Adobe Photoshop (version 2015.0.0) and OriginLab (version 9.0) respectively.

## 3. Results

### 3.1 Characterization

#### 3.1.1 FTIR

**Fig 2:**
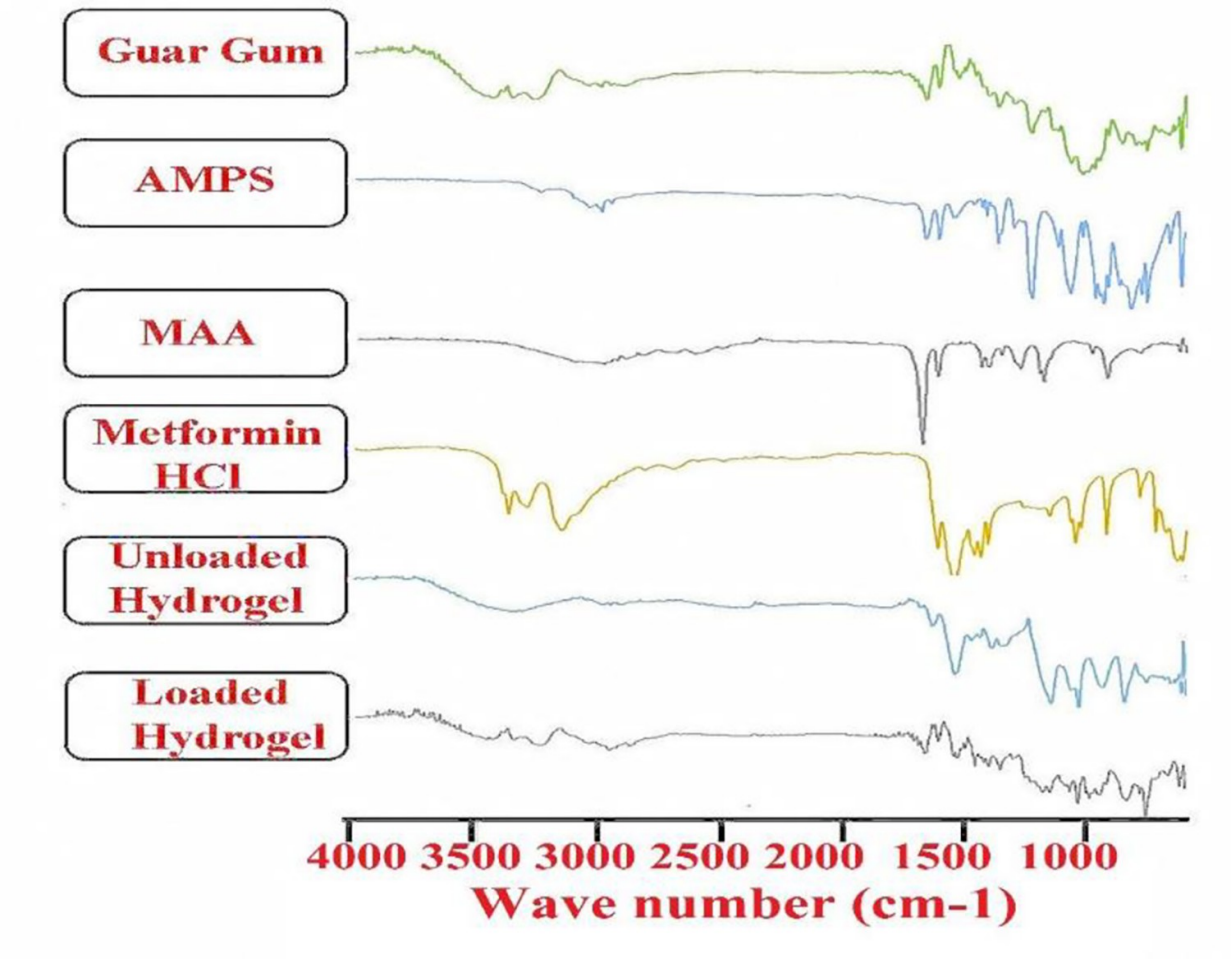
FTIR spectra of GG, AMPS, MAA, Metformin HCl, unloaded and drug-loaded hydrogels.

FTIR spectra of GG (Fig 2) displayed a broad peak in the range of 3900–3500 cm^-1^ which is associated to stretching of the–OH group. Peaks at 3251–2898 cm^-1^ are attributed to–CH and–NH groups respectively. Also, there is peak visible at 1371 cm^-1^ that represents C–O–C stretching vibrations of glyosidic linkage. Peaks in the range of 1026–788 cm^-1^ represents the existence of highly coupled C–C–O and C-OH and C-O-C in the polymer backbone [1].

In case of AMPS, peak at 2989 cm^−1^ is attributed to C-H stretching of–CH2 group. Peak at 1664 cm^−1^ is indicative of C = O stretching of amide-I group and peak at 1610 cm^−1^ (N–H bending of amide-II). Smaller band at 1370 and 1232 cm^−1^ represent asymmetric and symmetric stretching of S = O group respectively [24,25].

IR spectrum of methacrylic acid displayed some distinctive peaks. The peak at 2987 cm^−1^ identifies asymmetric stretching of the methyl group (C–H). Peak at 1698 cm^−1^ identified C = O stretching of COOH while peak at 1636 cm^−1^ recognizes the stretching vibrations of C = C [26].

Metformin hydrochloride spectrum (Fig 2) presents a sequence of stretching bands of–NH at 3368.2 cm^-1^ that is assigned to the primary amine, at 3293.62 cm^−1^ assigned to imine stretching, and at 3152.5 cm^−1^ assigned to secondary amine as revealed in Fig 2. At 1621.7 cm^−1^, there is bending vibration of primary–N while at 1562.16 cm^−1^ NH bending of imine group is shown. Bands between 1562 and 1417 cm^−1^ were owed by CH bending, and peaks at 1067, 936 and 800 cm^−1^ were assigned to N-H bending and C–N stretching [17,27].

The unloaded hydrogel discs showed peaks at 3500-3000cm^−1^ range indicating presence of -OH groups. As new bonds formed so the peaks appeared different from pure elements, similarly peaks at 3233, 2956 and 3457 cm^−1^ are clearly different from parent compounds. The 1075, 1460–1666 cm^−1^ peak range showed some sharp peaks confirming new bonds formation. New peaks were appeared in FTIR spectra of drug loaded Guar Gum-g-(AMPS-co-MAA) hydrogels at 1075 (N-H bending), 1460–1666 cm^−1^ (CH bending) which confirms the successful entrapment of drug into the polymeric network [17].

#### 3.1.2 X-ray diffraction analysis (XRD)

**Fig 3:**
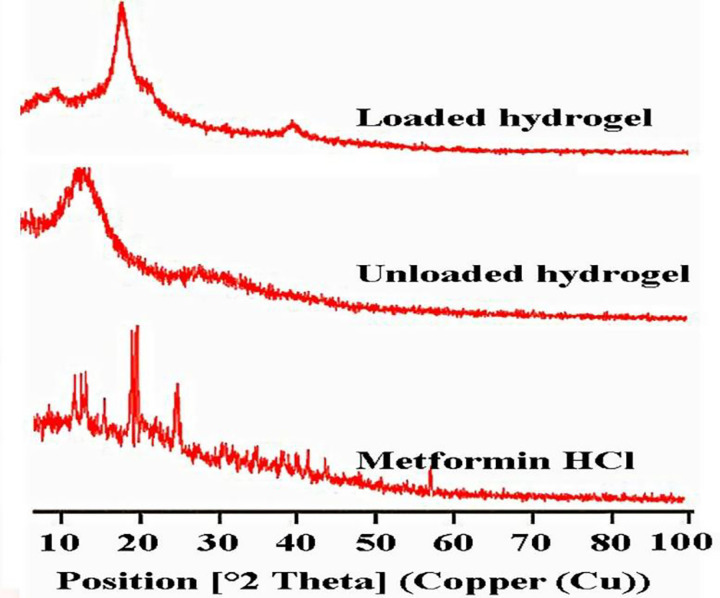
XRD patterns of Metformin HCl, unloaded and drug-loaded hydrogels.

It can be seen that Metformin HCl showed multiple sharp peaks at position 2θ **=** 15° to 45° thereby confirming the crystalline nature (Fig 3). The crystalline nature may be attributed to the existence of attraction forces as hydrogen bonding in the structure [28].

The XRD pattern of unloaded hydrogels showed a peak at position 2θ = 15^o^ and 30° confirming amorphous structure, whereas in drug loaded hydrogel, the peaks were disappeared showing the molecular dispersal of the drug in polymer matrix. The XRD pattern of hydrogel showed a wider peak at position 2θ = 20^o^ and 40^o^.

#### 3.1.3 Thermal gravimetric analysis (TGA)

**Fig 4:**
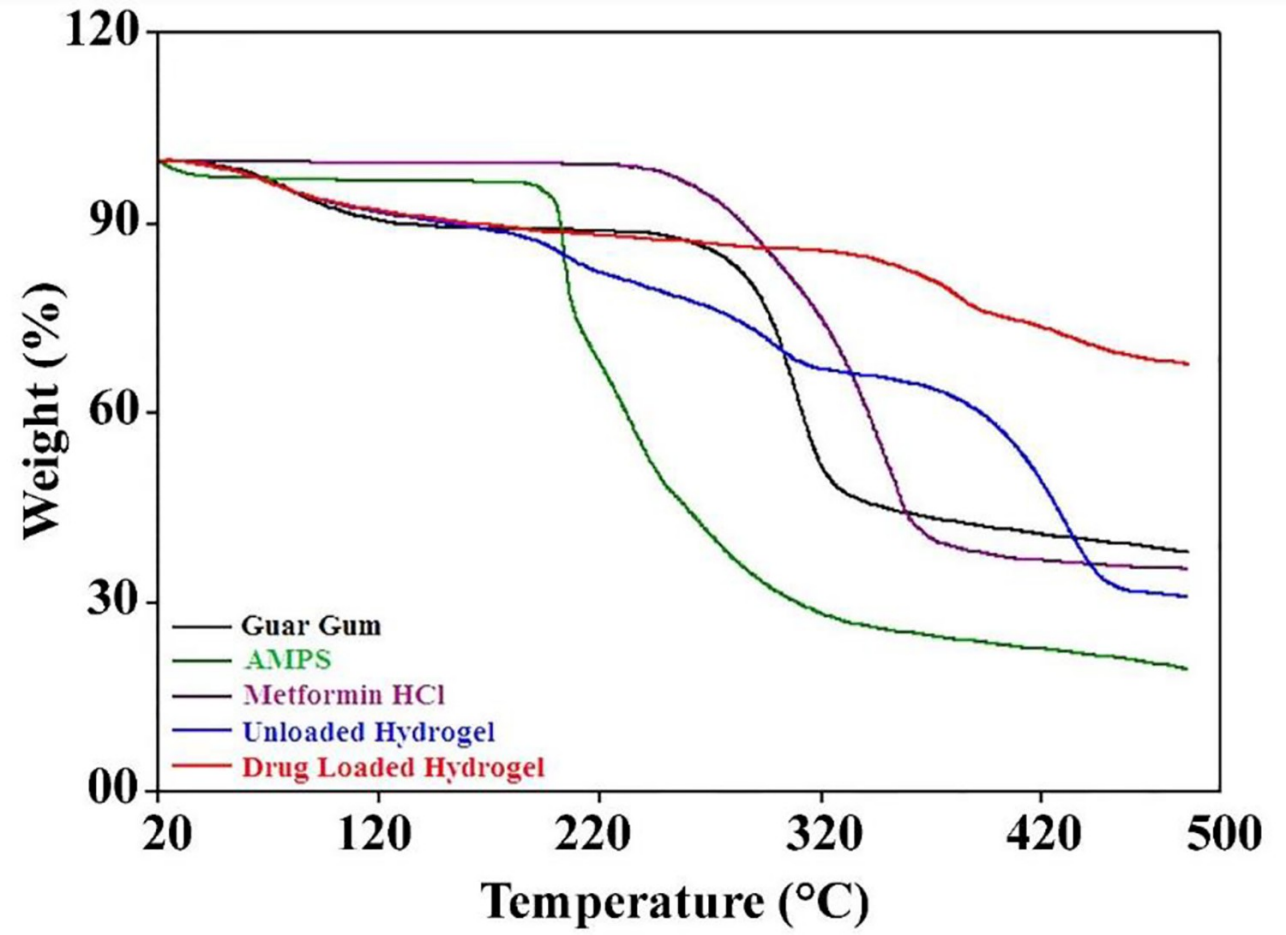
TGA curves of GG, AMPS, Metformin HCl, unloaded and drug-loaded hydrogels.

TGA profiles of GG, AMPS, drug unloaded and drug loaded hydrogels are shown in Fig 4 GG showed stability up to 210°C and degradation started thereafter. The initial minor weight loss around 240°C can be assigned to the removal of volatile components and moisture. In temperature range of 247°C to 330°C there was maximum weight loss (55%) which may be caused by degradation of polymer backbone [29].

AMPS displayed three stage weight loss. During first stage, approximately 30% weight was lost in the range of 55–220°C that could be assigned to water and moisture loss. The second stage was observed around 235°C contributing to 39.35% weight loss. This could be assigned to decomposition of SO_3_H group while the last stage around 330°C is mainly comprised of combustion of the degraded contents [30].

The TGA curve of metformin HCl showed initial weight loss at 258°C that is attributed to the release of NH_3_ molecule. Weight loss of 22.02% at 275.3°C is attributed to removal of hydrochloride molecule. The release of C_2_H_4_ molecule from the drug resulted in weight loss of 18% at 304.9°C while weight loss around 382°C could be assigned to decomposition of the drug [31].

In case of unloaded hydrogels, initial weight loss of approximately 12% in the range of 90–120°C can be attributed to water and moisture loss. The second phase in the temperature range of 230–320°C contributed for 24% lost may be attributed to decomposition of SO_3_H group of AMPS as well as initial decomposition of GG. Thereafter the weight loss up to 68.52% can be attributed to decomposition and combustion of network in the temperature range of 340–460°C. Drug loaded hydrogels displayed higher thermal stability in comparison with polymer, monomer, drug and unloaded hydrogels. The drug loaded hydrogel displayed minor weight loss of 9.71% up to 110°C. Thereafter no remarkable change was observed and hydrogel network was stable till 330°C with 10.83% weight loss. Pyrolysis and decomposition of network was observed above 350°C. The higher thermal stability may be attributed to successful crosslinking and drug loading.

#### 3.1.4 Differential scanning calorimetry (DSC)

**Fig 5:**
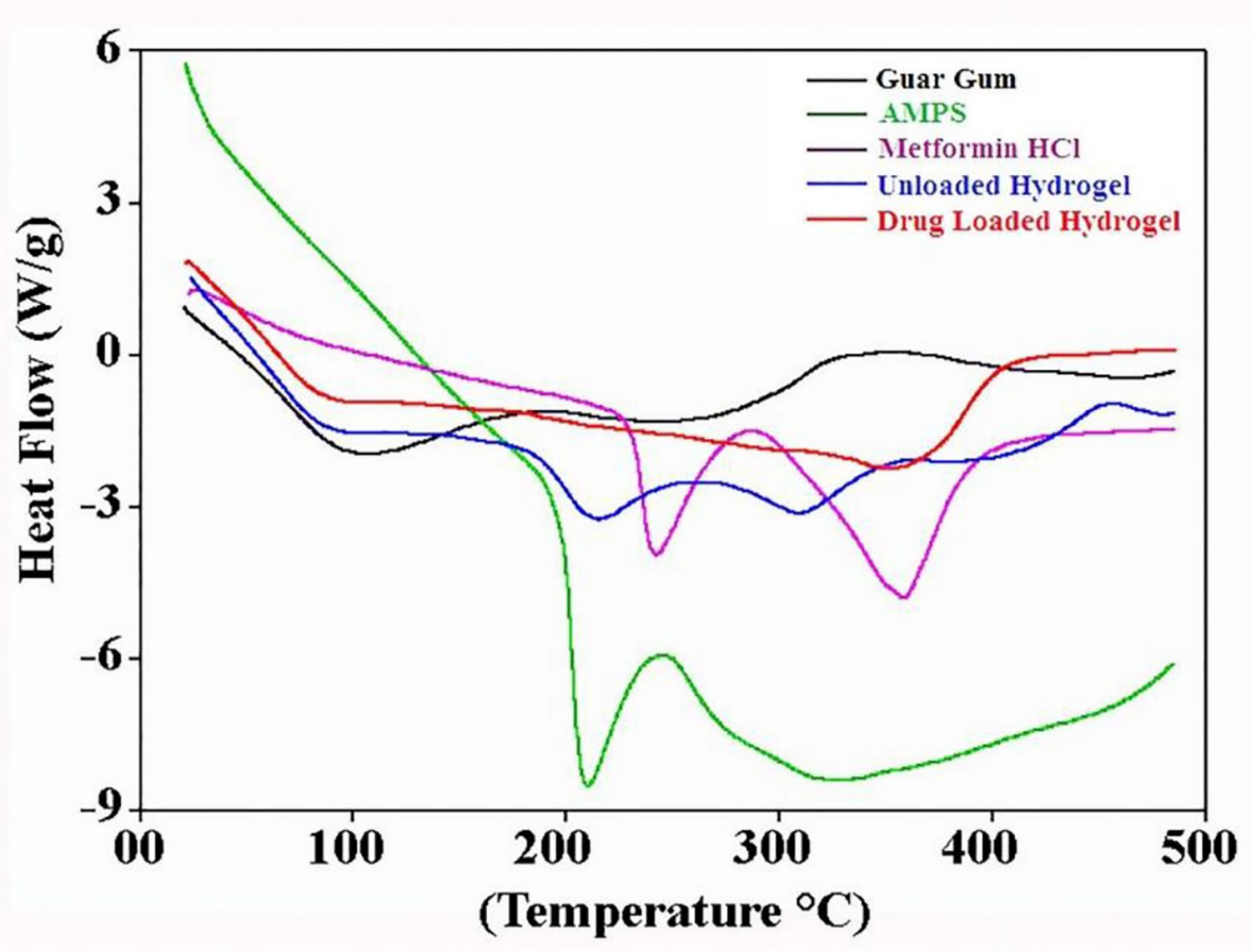
DSC curves of GG, AMPS, Metformin HCl, unloaded and drug-loaded hydrogels.

DSC thermogram (Fig 5) shows the thermal behavior of GG, AMPS, drug, unloaded and drug loaded hydrogels. The calorimetric profile of GG showed two endothermic peaks at 253°C and 296°C while exothermic peak at 317°C. The initial endothermic peak is assigned to early decomposition while second peak is attributed to initiation of combustion. The exothermic peak corresponds to decomposition (thermal and oxidative) of polymer, vaporization as well as elimination of volatile components. During pyrolysis of polysaccharides, there is randomized breakdown of glycosidic linkages followed by decomposition. Cleavage of galactose and mannose units are attributed to the wide decomposition profile of GG [32]. DSC thermogram of AMPS showed a sharp endothermic peak around 207°C that is attributed to decomposition of sulfonic acid group [5,20]. The thermal curve of pure metformin HCl exhibited two sharp endothermic peaks at 233°C and 372°C associated with fusion enthalpy indicating its anhydrous crystalline state [33,34].

Unloaded and drug loaded hydrogels displayed different thermal behavior in comparison with the individual components. In case of unloaded hydrogel, initial weight loss around 100°C is assigned to removal of water molecules. Slight endothermic peaks at 210°C and 305°C can be attributed to decomposition of SO_3_H group followed by decomposition of AMPS respectively. Drug loaded hydrogel showed initial weight loss due to removal of water and moisture contents in the range of 90–105°C while second endothermic peak at 370°C can be attributed to the decomposition of the drug. The new thermal behavior confirmed successful network formation and drug loading with enhanced thermal stability.

#### 3.1.5 Scanning electron microscopy (SEM)

**Fig 6:**
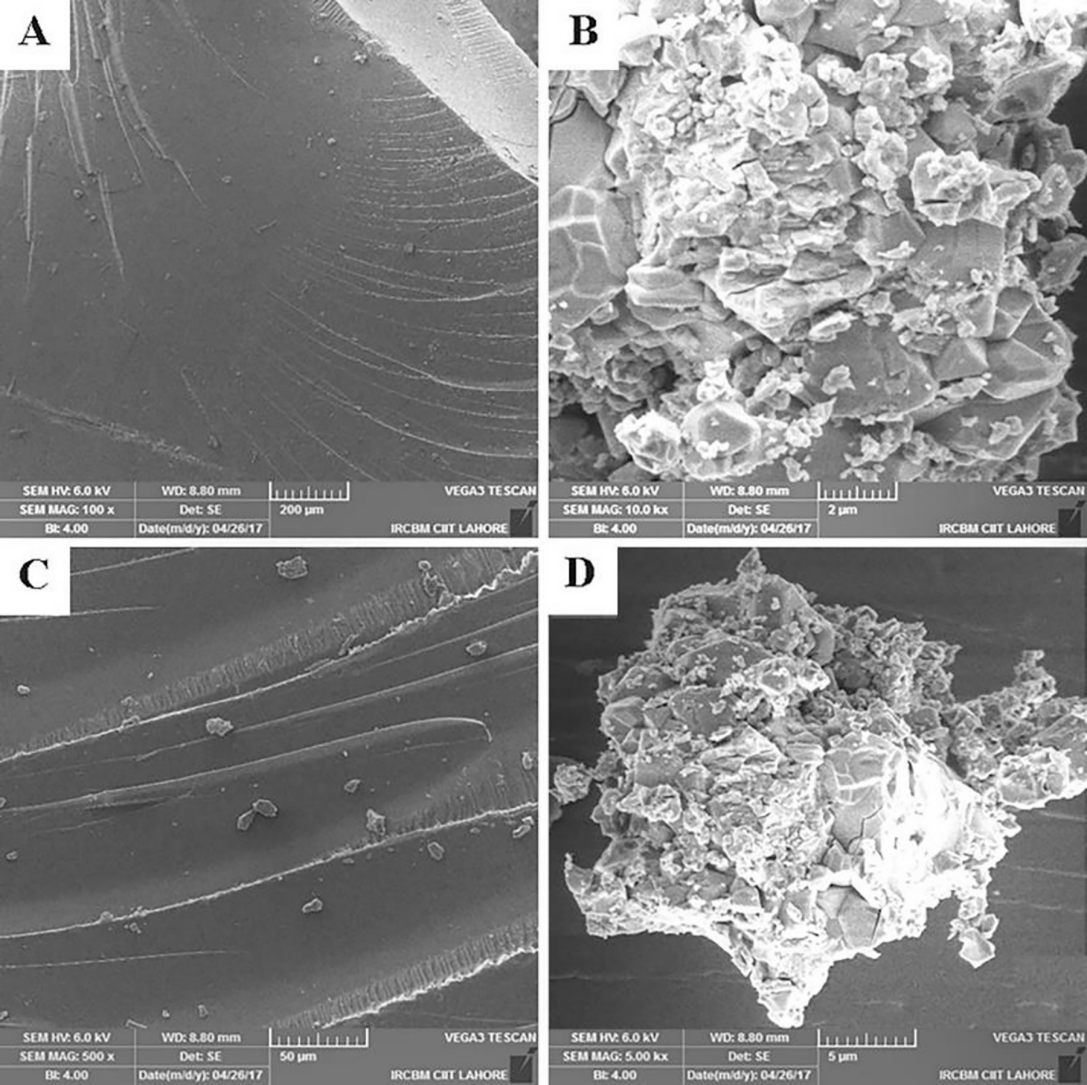
Surface micrographs (6-A and 6-C) of unloaded and drug-loaded hydrogels. Cross-sectional micrographs (6-B and 6-D) of unloaded and drug-loaded hydrogels respectively.

The surface morphology of unloaded and drug loaded hydrogel (Fig 6A and 6C respectively) demonstrated a clear and smooth surface of hydrogels and free of cracks with continuous and regular appearance. The cross-sectional view of both unloaded and drug loaded hydrogel (Fig 6B and 6D respectively) showed cracks with sharp edges, rough appearance and irregular shape.

### 3.2 Sol-gel analysis

Sol-gel analysis was performed to know about the un-crosslinked and crosslinked percent of the reactants. The effect of different feed ratios of GG, AMPS, and MAA is shown in S1 Table. The gel fraction was significantly increased by increasing the concentration of GG (p < 0.006), AMPS (p < 0.009) and MAA (p < 0.005). In general, gel fraction is increased by increasing the feed concentration of reactants that leads to provision of more active sites for completion of polymerization reaction. These results are in accordance with the previous study conducted by Ullah et al. while investigating pH sensitive hydrogels for colonic delivery of oxaliplatin [5,21]. Khanum et al. investigated AMPS based hydrogels and reported similar effect of reactants on gel fraction [35].

### 3.3 Drug entrapment efficiency (DEE)

Extraction by solvent replacement method was used to assess metformin HCl entrapment. According to the findings acquired, drug entrapment efficiency was decreased by increasing concentration of GG and AMPS likely due to decrease in swelling while MAA displayed inverse behavior as shown in Table 2.

**Table 2:** Percent metformin HCl entrapment and in-vitro release at pH 1.2, 6.8 and 7.4.

Formulation Code	Entrapped Metformin HCl (mg/Hydrogel Disc) ± SEM	% Metformin HCl Released		
		pH 1.2	pH 6.8	pH 7.4
GG1	74.6 ± 0.83	22.06	60.13	71.93
GG2	72.1 ± 0.91	17.44	66.91	71.25
GG3	69.3 ± 0.67	16.51	61.10	67.10
MA1	76.8 ± 0.46	21.45	69.26	76.22
MA2	77.6 ± 0.39	20.29	75.79	81.73
MA3	79.2 ± 0.81	36.19	77.37	89.20
AP1	68.9 ± 0.87	20.05	67.35	73.39
AP2	66.3 ± 0.26	18.64	65.72	70.38
AP3	65.6 ± 0.37	15.42	64.28	66.17

### 3.4 pH-responsiveness and metformin HCl release

#### 3.4.1 Effect of pH on swelling and drug release

The newly prepared hydrogels were exposed to various pH conditions in order to test its pH-responsiveness. In was observed that all the hydrogels have excellent pH-responsive behavior by displaying marked change in swelling and drug release. At low pH (1.2), low swelling and drug release was observed while with the increase in pH (6.8 and 7.4) there was significant increase in swelling and drug release for almost all formulations.

#### 3.4.2 Effect of GG on swelling and drug release

By increasing the feed concentration of GG (GG1-GG3), significant decrease in swelling behavior was observed at pH 1.2 (p < 0.003), pH 6.8 (p < 0.002) and pH 7.4 (p < 0.000). Similarly, drug release also followed the swelling pattern and there was significant decrease in drug release with increase in GG concentration at pH 1.2 (p < 0.007), pH 6.8 (p < 0.005) and pH 7.4 (p < 0.002) as shown in Table 2.

#### 3.4.3 Effect of MAA on swelling and drug release

Formulations with increased concentration of MAA (MA1-MA3) displayed significant decrease in swelling at pH 1.2 (p < 0.009) while significant increase in swelling was observed at pH 6.8 (p < 0.004) and pH 7.4 (p < 0.001). Similar trend was observed in drug release study and there was significant decrease at pH 1.2 (p < 0.005) while significant increase in drug release was displayed at pH 6.8 (p < 0.003) and pH 7.4 (p < 0.000) as shown in Table 2.

#### 3.4.4 Effect of AMPS on swelling and drug release

Increasing the concentration of AMPS (AP1-AP3) resulted in significant decrease in swelling at pH 1.2 (p < 0.007), pH 6.8 (p < 0.002) and pH 7.4 (p < 0.001). In case of drug release, similar trend was observed and there was decrease in drug release at pH 1.2 (p < 0.008), pH 6.8 (p < 0.009) and pH 7.4 (p < 0.004).

S1 Fig represent formulations (GG-1, MA-3 and AP-1) with highest swelling index and in-vitro drug release containing least amount of GG, AMPS and highest amount of MAA.

#### 3.4.5 Statistical and comparative analysis of swelling, drug loading and drug release data

Swelling data was analyzed using ANOVA (one factor) that confirmed significant difference between swelling index of GG and MAA (p < 0.003), GG and AMPS (p < 0.002) and MAA and AMPS (p < 0.005) at pH 7.4. Similarly, significant difference was observed between percent drug release of GG and MAA (p < 0.005), GG and AMPS (p < 0.003) and MAA and AMPS (p < 0.007) at pH 7.4. In case of drug loading, significant decrease was observed with increase in concentration of GG (p < 0.03), MAA (p < 0.05) and AMPS (p = 0.009). By comparing the overall drug entrapment efficiency, significant difference was observed between GG and MAA (p < 0.02), GG and AMPS (p < 0.009) and MAA and AMPS (p < 0.002).

### 3.5 Metformin HCl release kinetics

S2 Table shows metformin HCl release kinetics. By fitting the in-vitro drug release to some kinetic models, it was found the metformin HCl release data best fit the Zero-Order (concentration independent) for almost all the formulations. Value of diffusion co-efficient (n) lies between 0.5 and 0.85.

### 3.6 Oral tolerability studies

#### 3.6.1 Animal procurement

Among the initially procured 16 rabbits (08 male and 08 female), 04 rabbits were eliminated from the final study as 2 rabbits were overweight (2.5 kg), 01 was under weight (1.7 kg) and 01 rabbit showed signs of illness. Overall 12 rabbits were procured for the final study as this number was sufficient according to the criteria proposed by laboratory animal’s science association and FDA.

#### 3.6.2 Maximum tolerance dose of hydrogel suspension

For MTD calculation, 4 rabbits (02 male and 02 male) were selected separately and hydrogel suspension was administered in the range of 400–3800 mg/kg of body weight. At 3800 mg/kg dose, rabbits showed signs like loss of appetite, decreased water intake and increased salivation possibly caused by stomach blotting. In the view of these signs, 3600 mg/kg of hydrogel suspension was finalized as maximum dose for administration.

#### 3.6.3 Monitoring of vital signs and symptoms

During the entire course of study, no toxicity or mortality was observed among the test and control group rabbits. Salivation, lacrimation, breathing, urination, and response to sound and light were normal at the dose of 3600 mg/kg of body weight. Beside this, feces color was normal, free of pus and blood. Food and water intake of both the control and treatment group was almost the same.

#### 3.6.4 Hematology and serum chemistry

It is possible that the unreacted contents of hydrogel system may leach out and cause any abnormality in a biological system after oral administration. Blood samples of both the groups were collected and tested for hematology and serum chemistry in order to investigate any toxic or abnormal effect.

Tables 3 and 4 represent the hematology and serum chemistry data of both the control and treatment groups. The results obtained from treatment group suggest normal kidney and liver function as well as normal blood systems as the values are fairly in resemblance with the obtained results for control group. No significant difference was observed between the metabolite concentrations of both the groups. Serum chemistry and hematology results suggest that the fabricated hydrogel system was safe on the basis of its composition as well as the administered dose.

**Table 3:** Biochemical analysis.

Biochemical Analysis	Group-I (Control)		Group-II (Treatment)	
	Male (n = 03)	Female (n = 03)	Male (n = 03)	Female (n = 03)
pH	6.92 ± 0.10	7.03 ± 0.10	7.14 ± 0.06	7.12 ± 0.08
Hemoglobin (g/L)	113.67 ± 1.53	107.33 ± 1.53	115.67 ± 1.43	108.67 ± 1.50
RBCs × 10^12^/L	6.03 ± 0.10	5.70 ± 0.21	6.15 ± 0.08	6.12 ± 0.09
Eosinophils × 10^9^/L	8.02 ± 0.08	8.12 ± 0.11	7.97 ± 0.15	8.14 ± 0.06
WBCs × 10^9^/L	0.04 ± 0.01	0.03 ± 0.01	0.04 ± 0.01	0.04
Lymphocytes ×10^9^/L	2.28 ± 0.08	2.26 ± 0.07	2.68 ± 0.04	2.40 ± 0.07
Neutrophils × 10^9^/L	4.22 ± 0.05	4.43 ± 0.12	4.31 ± 0.03	4.29 ± 0.08
Basophils × 10^9^/L	0.38 ± 0.04	0.31 ± 0.04	0.35 ± 0.02	0.38 ± 0.03
Platelets × 10^9^/L	348.29 ± 6.29	337.00 ± 3.29	355.67 ± 3.79	349.33 ± 0.71
PCV (L/L)	0.36 ± 0.01	0.32	0.43 ± 0.02	0.44 ± 0.01
MCV (L/L	66.80 ± 1.49	64.49 ± 1.54	68.77 ± 1.91	66.03 ± 1.01

PCV* for packed cell volume, MCV* for mean cell volume.

**Table 4:** Hematological analysis.

Parameter	Group-I (Control)		Group-II (Treatment)	
	Male (n = 03)	Female (n = 03)	Male (n = 03)	Female (n = 03)
Total Protein (g/L)	70.83 ± 1.35	72.09 ± 1.93	71.47 ± 0.87	70.04 ± 0.39
Globulin (g/L)	22.23 ± 2.65	20.81 ± 1.76	23.11 ± 1.83	21.88 ± 0.40
Albumin (g/L)	50.17 ± 1.26	52.55 ± 1.37	53.12 ± 1.14	51.66 ± 0.55
ALT (U/L)	80.22 ± 2.68	78.46 ± 1.36	79.63 ± 1.71	80.16 ± 0.23
ALP (U/L)	134.03 ± 2.63	127.23 ± 1.19	130.64 ± 1.65	131.10 ± 2.30
AST (U/L)	60.67 ± 2.26	57.17 ± 2.56	61.34 ± 0.78	59.08 ± 1.17
Cholesterol (mmol/L)	112.63 ± 1.80	105.51 ± 2.67	110.95 ± 1.38	106.61 ± 1.76
Glucose (mmol/L)	8.27 ± 0.90	7.04 ± 0.21	7.73 ± 0.38	7.71 ± 0.33
Creatinine (μmol/L)	153.99 ± 1.50	156.51 ± 2.30	152.98 ± 0.98	157.35 ± 1.58
Urea (mmol/L)	15.07 ± 1.17	17.10 ± 0.55	17.07 ± 0.92	17.37 ± 0.46
Uric Acid (mg/dL)	3.28 ± 0.23	3.76 ± 0.16	3.49 ± 0.07	3.90 ± 0.12
Magnesium (mmol/L)	0.72 ± 0.07	0.69 ± 0.03	0.79 ± 0.05	0.74 ± 0.06
Phosphorus (mmol/L)	2.62 ± 0.18	2.73 ± 0.22	2.84 ± 0.10	2.68 ± 0.03
Potassium (mmol/L)	5.94 ± 0.28	6.25 ± 0.24	5.66 ± 0.16	6.36 ± 0.09
Sodium (mmol/L)	156.81 ± 1.88	148.30 ± 1.87	152.22 ± 1.87	146.38 ± 0.94

ALT* for alanine aminotransferase, ALP* for alkaline phosphatase, AST aspartate aminotransferase.

#### 3.6.5 Histopathological examination

**Fig 7:**
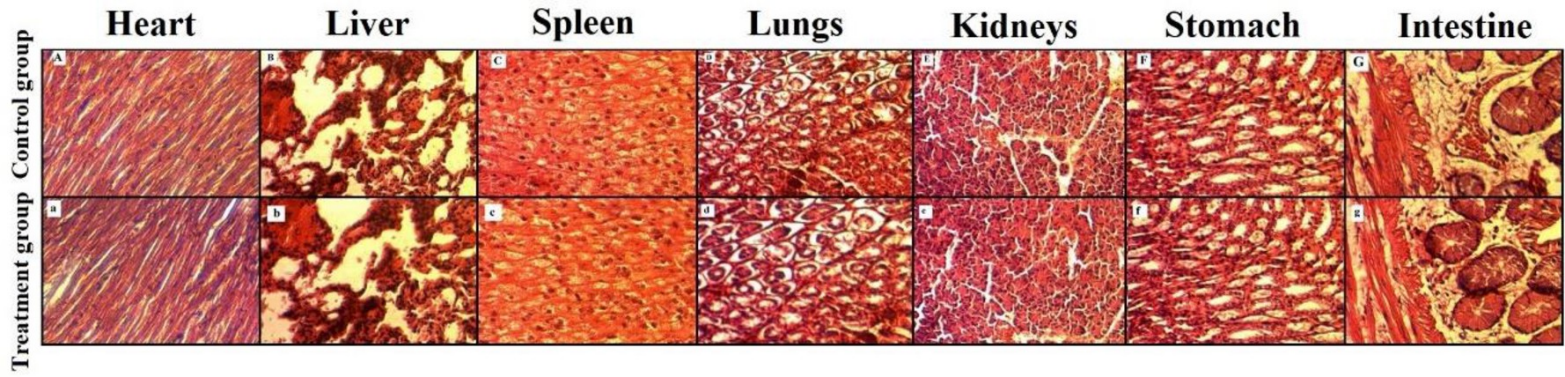
Histopathological micrographs of heart, liver, spleen, lungs, kidneys, stomach and intestine of control and treatment groups.

The Histopathological micrographs of heart, lungs, liver, stomach, spleen, kidney and intestine of treatment and control group are presented in Fig 7.

There was no significant difference between the cardiac muscles and cardiac myocytes of treated and control group without any hypertrophy. There was no abnormality in pericardium, myocardium and endocardium.

In case of liver, no signs of necrosis or degeneration were observed. Hepatic sinusoid displayed no signs of hypertrophy, neutrophil, macrophages, lymphocytes infiltration or hyperemia. Additionally, hepatic lobules and cord were found in normal order. Histopathological micrographs of spleen displayed normal corpuscle structure without any gross difference in red pulp, white pulp and sinus. No alveolar or bronchial collapse was found in the lung micrographs of both the control and treatment group. Surroundings of the bronchus were normal and no infiltration of inflammatory cells was observed. Additionally, cilia were intact and airway was clear and normal. In case of kidneys, shape of nephron was normal with defined space around the glomerulus. No signs of bleeding, necrosis or degeneration were observed in glomeruli and kidney tubes. Adrenal cortex and medulla were found normal. There were no signs of inflammation, bleeding or ulceration on the mucosal lining of stomach. Intestinal lining were found normal and there were no signs of bleeding, degeneration or necrosis.

## 4. Discussion

pH-responsive GG-MAA-AMPS hydrogels were prepared following free radical polymerization technique. The newly prepared hydrogels were characterized through FTIR, XRD, TGA, DSC and SEM. FTIR results confirmed the successful crosslinking of reactants and drug loading without structural alteration of polymer, monomer and drug. The characteristics peaks of polymer, monomers and drug were present in unloaded and drug-loaded hydrogel with slight shifting (30). XRD patterns of drug loaded hydrogels indicated decrease in crystallinity of structure, which means that when drug was loaded in hydrogel it retained its structure but crystallinity decreased due to decreased electrostatic attractions and strong filling of hydrogel spaces with drug molecules. This can be attributed to breaking of inter and intramolecular attraction forces as result of new bonds formation [36]. In case of TGA and DSC, higher thermal stability was observed for unloaded and drug-loaded hydrogels in comparison with the reactants that also confirmed successful crosslinking and drug loading. The gel fraction was increased with the increase in feed concentration of reactants that leads to provision of more active sites for completion of polymerization reaction. Our obtained results are in accordance with the previous study conducted by Ullah et al. while investigating pH sensitive hydrogels for colonic delivery of oxaliplatin [5,21]. Khanum et al. investigated AMPS based hydrogels and reported similar effect of reactants on gel fraction [35].

The pH of the medium and acidic components of the polymer play an important role in swelling and drug release behavior of the hydrogels. Efficient swelling is attained when the pKa value of the components of the buffer is above the pKa value of the carboxylic groups of the gel. In such conditions, the gel will ionize and protons will be accepted by the buffer. With the increase in pH,–COOH groups were protonated that resulted in ionic repulsion and polymer chain relaxation. The relaxed polymeric chain enable high water penetration and thus high osmotic pressure is built leading to increased swelling [37]. On the other hand, decrease in pH resulted in decreased swelling caused by the conversion of highly ionized COO- groups to COOH groups. As a result of this conversion, ionic groups were ionized followed by precipitation and shrinkage of polymeric network [6]. Similar trend was observed in drug release experiment. There was an increase in drug release with increase in pH. As pH was increased, there was increased swelling as well as increased osmotic pressure inside polymeric network that resulted in increased drug release.

By increasing the concentration of GG, there was decrease in swelling, drug loading and drug release.

GG is comprised of two fold guaran helix that is stabilized by intramolecular hydrogen bonds between mannan main chain and galactosyl side chains. These hydrogen bonds are present in periodic pattern. Upon crosslinking with MAA there may be intermolecular hydrogen bond formation that in turn will affect the swelling behavior of GG. With the increase in concentration of GG, there will be increased hydrogen bond formation between GG and MAA thereby reducing the hydrogen bond formation with water. Additionally, increase in GG concentration will result in dense polymeric network formation leading to reduced water diffusion and also polymer chain relaxation. Previously, Das & Subuddhi, 2015, investigated GG based pH-responsive hydrogels for intestinal delivery of dexamethasone and reported similar effect of GG on swelling and drug release [6]. similarly, Li et al., 2006 investigated guar gum/poly (acrylic acid) hydrogels and reported similar effect of guar gum [38].

As the concentration of MAA is increased, there is increase in overall percent grafting on polymer backbone due to presence of increased number of MAA molecules. This also leads to development of hydrogels with increased density, crosslinking and percentage of elastic polymer chains. MAA being ionic in nature responds to variations in pH of surrounding environment. At low pH, there is decreased swelling because the COOH groups of MAA remains protonated resulting in collapsed polymer network with less osmotic pressure. With the increase in pH above pKa of MAA, deprotonation takes place and COOH groups are ionized to negatively charge–COO ions. These–COO ions result in increased repulsion, increased osmotic pressure and hence increased swelling and drug release. Our obtained results are in accordance with the study reported by Haider et al., while investigating chitosan-MAA supramolecular hydrogels for polyoxometalate [37]. Similarly, Ullah et al., investigated pectin-LA-MAA pH-responsive hydrogels for colonic delivery of oxaliplatin and reported similar effect of MAA on swelling and drug release [2].

This decrease in swelling and drug release with the increase in concentration of AMPS is attributed to presence of active SO_3_H groups that interact with the drug molecules leading to drug release retardation. Additionally, increase in AMPS concentration results in formation of dense network which in turn reduce the transport of monomeric molecules and free radicals. Previously, Khanum et al., reported similar effect of AMPS on swelling and drug release in HMPC-co-AMPS hydrogels for controlled delivery of loxoprofen sodium [35]. Similarly, Anwaer et al., synthesized alginate-PVA-AMPS hydrogels for tramadol HCl and reported similar effect of AMPS on swelling and drug release [39].

Diffusion, erosion of polymeric network and aqueous solubility of the drug are among the key factors that play significant role in releasing the drug from polymer based drug delivery system. Mostly, the hydrophilic drugs follow a specific release pattern that involve entry of the surrounding dissolution medium via micro-channels of polymeric network followed by dissolution of the drug and subsequent movement of the dissolution medium containing dissolved molecules of the drug to the surface and then periphery [40]. S2 Table shows metformin HCl release kinetics. By fitting the in-vitro drug release to some kinetic models, it was found the metformin HCl release data best fit the Zero-Order (concentration independent) for almost all the formulations indicating the pre-dominant effect of polymeric network relaxation of drug release. Almost all the formulations displayed extensive swelling in short time that may be attributed to rapid relaxation of polymeric network followed by buffer diffusion [6]. Thus, it is evident that metformin HCl release from these hydrogels is mainly controlled by relaxation rate of the polymeric network chains. Value of diffusion co-efficient (n) lies between 0.5 and 0.85 indicating the anomalous nature of metformin HCl release, where the process is contributed by both diffusion as well as polymeric network relaxation.

Oral tolerability studies showed promising results and conclusively, there was no gross difference between histo-pathological micrographs of control and treatment groups. Our obtained results of hematology, serum chemistry and histopathology are well supported by findings of Barkat et al., while studying pH-responsive hydrogels for colonic delivery of oxaliplatin [41]. Similarly, our obtained results were in close relationship with the previous study conducted by Ullah et al., while examining the fabrication and evaluation of pH-responsive hydrogels [2].

## Conclusion

In conclusion, a series of GG-MAA-AMPS based pH-responsive hydrogels were prepared by free radical polymerization technique. The interaction of polymers, thermal behavior, crystallinity profile and morphology evaluation were performed using advanced characterization techniques. FTIR analysis revealed the successful crosslinking of GG, MAA and AMPS as well as drug loading. XRD patterns displayed decrease in crystallinity Metformin HCl after loading in newly prepared hydrogel system. TGA and DSC results demonstrated the higher thermal stability of hydrogels in comparison with GG, MAA and AMPS. Sol-gel analysis confirmed that gel content was increased with the increase in feed ratio of GG, MAA and AMPS. The prepared hydrogels displayed low swelling and drug release at pH 1.2 while high at pH 6.8 and 7.4 thus proved to be pH dependent. Decrease in swelling and drug release was observed by increasing the feed ratio of GG and AMPS while MAA acted inversely. Kinetic modeling confirmed that drug release followed Zero-order kinetics while release mechanism was based on polymeric network relaxation and solvent diffusion. Oral tolerability study showed that hydrogel suspension was well tolerable up to 3600 mg/kg of body weight without causing hematological or histo-pathological changes. Based on the obtained results, the prepared hydrogels can be suggested as a potential carrier for oral-colonic delivery of metformin HCl as well as other hydrophilic drugs.

## Supporting information

S1 Fig Formulations of guar gum (GG-1), MAA (MA-3) and AMPS (AP-1) with highest swelling and drug release ratio. (TIF)

S1 Table Effect of GG, MAAA and AMPS feed concentrations on gel fraction. (PDF)

S2 Table Kinetic modeling of in-vitro drug release. (PDF)
