# Supplementary figures and images for: Development, characterization and In-vitro evaluation of guar gum based new polymeric matrices for controlled delivery using metformin HCl as model drug
Source: PLoS One. 2022 Jul 28;17(7):e0271623. doi: 10.1371/journal.pone.0271623 (PMC9333214; doi:10.1371/journal.pone.0271623)

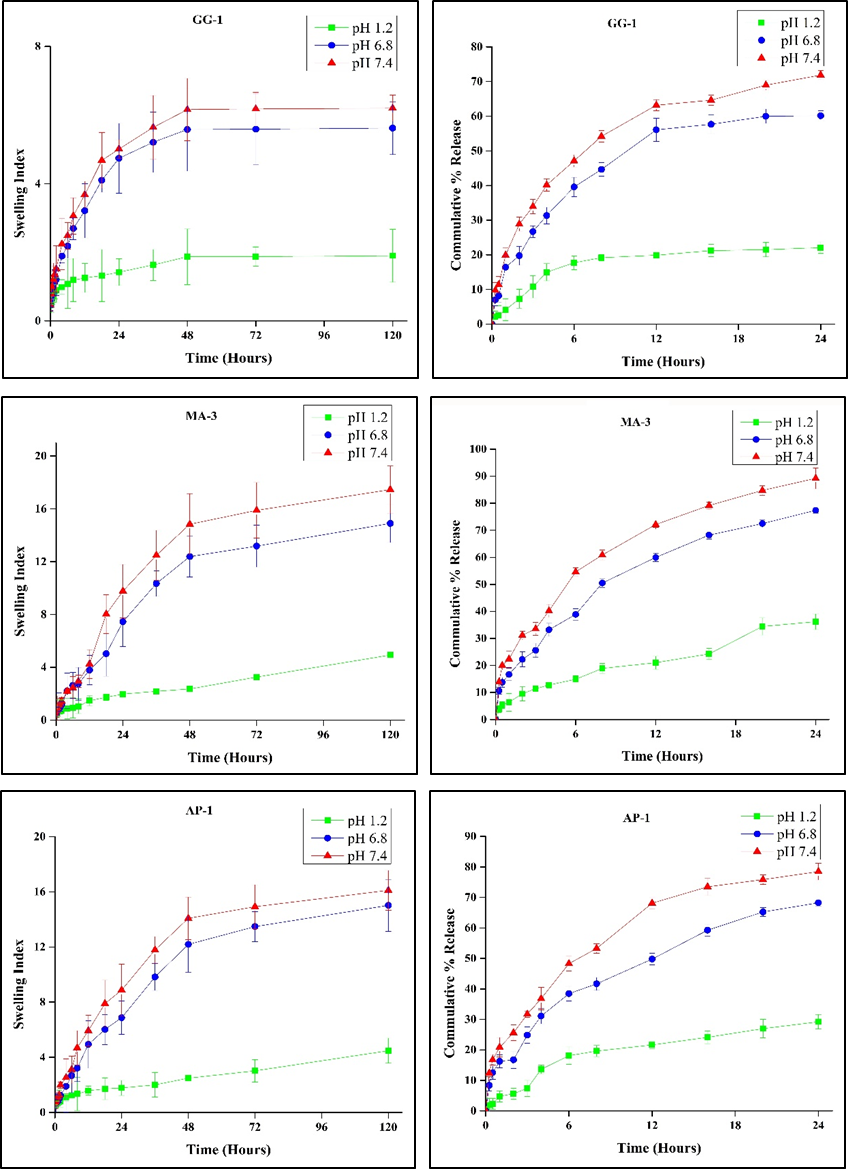

Supplement: S1 Fig — (TIF) [file pone.0271623.s001.tif]
