# Supplementary material for: Development, characterization and In-vitro evaluation of guar gum based new polymeric matrices for controlled delivery using metformin HCl as model drug
Source: PLoS One. 2022 Jul 28;17(7):e0271623. doi: 10.1371/journal.pone.0271623 (PMC9333214; doi:10.1371/journal.pone.0271623)
Supplement: S1 Table — (PDF) [file pone.0271623.s002.pdf]

Formulation Code	Sol Fraction (%)	Gel Fraction (%)
GG-1	14.46 ± 0.043	85.54 ± 0.43
GG-2	11.62 ± 0.021	88.38 ± 0.021
GG-3	5.69 ± 0.016	94.31 ± 0.16
MA-1	16.77 ± 0.003	83.27 ± 0.003
MA-2	12.37 ± 0.039	87.63 ± 0.039
MA-3	7.55 ± 0.042	92.45 ± 0.042
AP-1	14.75 ± 0.011	85.25 ± 0.011
AP-2	10.33 ± 0.018	89.67 ± 0.018
AP-3	5.87 ± 0.022	94.13 ± 0.022

GG* for guar gum, MA* for methacrylic acid, AP* for 2-acrylamido 2-methylprone sulfonic acid.
