# Supplementary material for: Development, characterization and In-vitro evaluation of guar gum based new polymeric matrices for controlled delivery using metformin HCl as model drug
Source: PLoS One. 2022 Jul 28;17(7):e0271623. doi: 10.1371/journal.pone.0271623 (PMC9333214; doi:10.1371/journal.pone.0271623)
Supplement: S2 Table — (PDF) [file pone.0271623.s003.pdf]

Formulation Code	pH	R^2				Value of "n"
		Zero-Order	First-Order	Higuchi Model	Korsmeyer-Peppas Model	
GG-1	1.2	0.762	0.825	0.899	0.847	0.617
	6.8	0.695	0.813	0.840	0.831	0.710
	7.4	0.668	0.804	0.819	0.778	0.615
GG-2	1.2	0.752	0.804	0.822	0.823	0.633
	6.8	0.695	0.823	0.772	0.812	0.593
	7.4	0.686	0.848	0.760	0.747	0.717
GG-3	1.2	0.892	0.922	0.874	0.838	0.618
	6.8	0.783	0.847	0.810	0.820	0.686
	7.4	0.733	0.833	0.798	0.758	0.612
MA-1	1.2	0.796	0.922	0.842	0.825	0.767
	6.8	0.742	0.901	0.812	0.714	0.850
	7.4	0.715	0.894	0.801	0.697	0.564
MA-2	1.2	0.777	0.909	0.848	0.840	0.728
	6.8	0.726	0.888	0.870	0.774	0.734
	7.4	0.712	0.844	0.885	0.741	0.636
MA-3	1.2	0.839	0.957	0.929	0.893	0.527
	6.8	0.815	0.915	0.944	0.876	0.729
	7.4	0.792	0.903	0.955	0.849	0.847
AP-1	1.2	0.813	0.830	0.864	0.849	0.595
	6.8	0.841	0.878	0.880	0.828	0.869
	7.4	0.853	0.899	0.875	0.801	0.719
AP-2	1.2	0.830	0.934	0.965	0.901	0.621
	6.8	0.790	0.947	0.939	0.832	0.668
	7.4	0.748	0.959	0.911	0.806	0.722
AP-3	1.2	0.893	0.957	0.937	0.864	0.604
	6.8	0.776	0.867	0.890	0.803	0.775
	7.4	0.723	0.826	0.833	0.754	0.526

GG* for guar gum, MA* for methacrylic acid, AP* for 2-acrylamido 2-methylprone sulfonic acid.
